# Assessing Causal Pathways and Targets of Implementation Variability for EBP use (Project ACTIVE): a study protocol

**DOI:** 10.1186/s43058-021-00245-3

**Published:** 2021-12-20

**Authors:** Emily M. Becker-Haimes, David S. Mandell, Jessica Fishman, Nathaniel J. Williams, Courtney Benjamin Wolk, Katherine Wislocki, Danielle Reich, Temma Schaechter, Megan Brady, Natalie J. Maples, Torrey A. Creed

**Affiliations:** 1 Department of Psychiatry, University of Pennsylvania, Perelman School of Medicine, 3535 Market Street, 3rd floor, Philadelphia, PA 19104 USA; 2 Hall Mercer Community Mental Health, University of Pennsylvania Health System, Philadelphia, PA USA; 3 Message Effects Laboratory, Annenberg School for Communication, University of Pennsylvania, Philadelphia, PA USA; 4 School of Social Work, Boise State University, Boise, ID USA; 5 Department of Psychiatry and Behavioral Sciences, University of Texas Health Science Center at San Antonio, San Antonio, TX USA

**Keywords:** Implementation science, Causal model, Cognitive-behavioral therapy, Community mental health, Behavior change theory, Theory of planned behavior, Organizational theory

## Abstract

**Background:**

Advancing causal implementation theory is critical for designing tailored implementation strategies that target specific mechanisms associated with evidence-based practice (EBP) use. This study will test the generalizability of a conceptual model that integrates organizational constructs and behavioral theory to predict clinician use of cognitive-behavioral therapy (CBT) techniques in community mental health centers. CBT is a leading psychosocial EBP for psychiatric disorders that remains underused despite substantial efforts to increase its implementation.

**Methods:**

We will leverage ongoing CBT implementation efforts in two large public health systems (Philadelphia and Texas) to recruit 300 mental health clinicians and 600 of their clients across 40 organizations. Our primary implementation outcomes of interest are clinician intentions to use CBT and direct observation of clinician use of CBT. As CBT comprises discrete components that vary in complexity and acceptability, we will measure clinician use of six discrete components of CBT. After finishing their CBT training, participating clinicians will complete measures of organizational and behavior change constructs delineated in the model. Clinicians also will be observed twice via audio recording delivering CBT with a client. Within 48 h of each observation, theorized moderators of the intention-behavior gap will be collected via survey. A subset of clinicians who report high intentions to use CBT but demonstrate low use will be purposively recruited to complete semi-structured interviews assessing reasons for the intention-behavior gap. Multilevel path analysis will test the extent to which intentions and determinants of intention predict the use of each discrete CBT component. We also will test the extent to which theorized determinants of intention that include psychological, organizational, and contextual factors explain variation in intention and moderate the association between intentions and CBT use.

**Discussion:**

Project ACTIVE will advance implementation theory, currently in its infancy, by testing the generalizability of a promising causal model of implementation. These results will inform the development of implementation strategies targeting modifiable factors that explain substantial variance in intention and implementation that can be applied broadly across EBPs.

Contributions to the literature

- Understanding the causal, multilevel pathways that influence evidence-based practice implementation is critical for designing effective implementation strategies; however, to date, empirical tests of causal implementation pathways have been limited.
- This study will fill this gap by testing a promising model that integrates organizational and behavioral change theories from leading implementation science frameworks to predict the implementation of cognitive-behavioral therapy techniques in a sample of 300 clinicians and 600 clients.
- Results will identify promising predictive pathways and whether pathways differ as a function of the specific evidence-based practice being implemented to guide the generation of tailored and potentially more effective implementation strategies.

## Background

Recent research documents the high cost of many implementation strategies [1] and their relatively modest effects on increasing clinician use of evidence-based practices (EBPs) to improve mental health [2–5]. Tailored implementation strategies, which target specific mechanisms associated with the use of specific EBP components, may be more successful and efficient than general implementation strategies in facilitating clinician behavior change [6, 7]. Tailoring strategies requires identifying the specific mechanisms that affect implementation success. Unfortunately, the current understanding of the mechanistic processes by which implementation strategies affect clinician behavior change is poor [6], and often relies on small samples and qualitative or mixed methods [8], which limits the generalizability of findings. Furthermore, an empirical study is often limited to examining the associations among self-reported implementation outcomes and a select number of candidate implementation determinants, which may or may not be mutable [9]. Elucidating the mutable causal processes underlying EBP implementation using prospective data collection and objective measurement of implementation outcomes is a critical next step for advancing implementation theory and practice.

This study will test causal pathways of implementation guided by a theoretical model that integrates organizational theory [10] and behavioral science [11]. The model aims to predict clinician use of six distinct components of cognitive-behavioral therapy (CBT), a leading psychosocial EBP for psychiatric disorders [12]. Integrating organizational and behavioral prediction theory and anchoring causal modeling around clinician intentions is a promising approach to building causal models in implementation science. *Implementation frameworks* describe the complex, multilevel variables that influence clinician behavior, yet rarely posit causal mechanisms [6, 13]. *Behavior change theories* describe the causal chains that lead to human behavior change (in our case, EBP uptake), yet generally do not account for the complex, multilevel processes that affect implementation [14, 15]. *Intentions*, which reflect one’s level of motivation to perform a behavior, are a validated, useful construct around which to build causal models of implementation and identify malleable mechanisms of implementation. Strong intentions will be followed by changes in behavior if the individual has the skills and resources needed to perform the given behavior [16–18]. Thus, studying intentions facilitates the study of mechanisms that influence both intention formation (i.e., antecedent factors influencing clinicians’ intentions to use an EBP, such as clinician attitudes, subjective norms, and self-efficacy) and moderators of the intention-behavior gap (i.e., factors that facilitate or impede an individual clinician from acting on their intention to use an EBP) [19]. Previous work has demonstrated the feasibility and utility of incorporating organizational theory with behavioral theory to successfully predict as much as 75% of the variance in teachers’ implementation of EBPs for children with autism [20].

### Objectives and aims

**Fig. 1:**
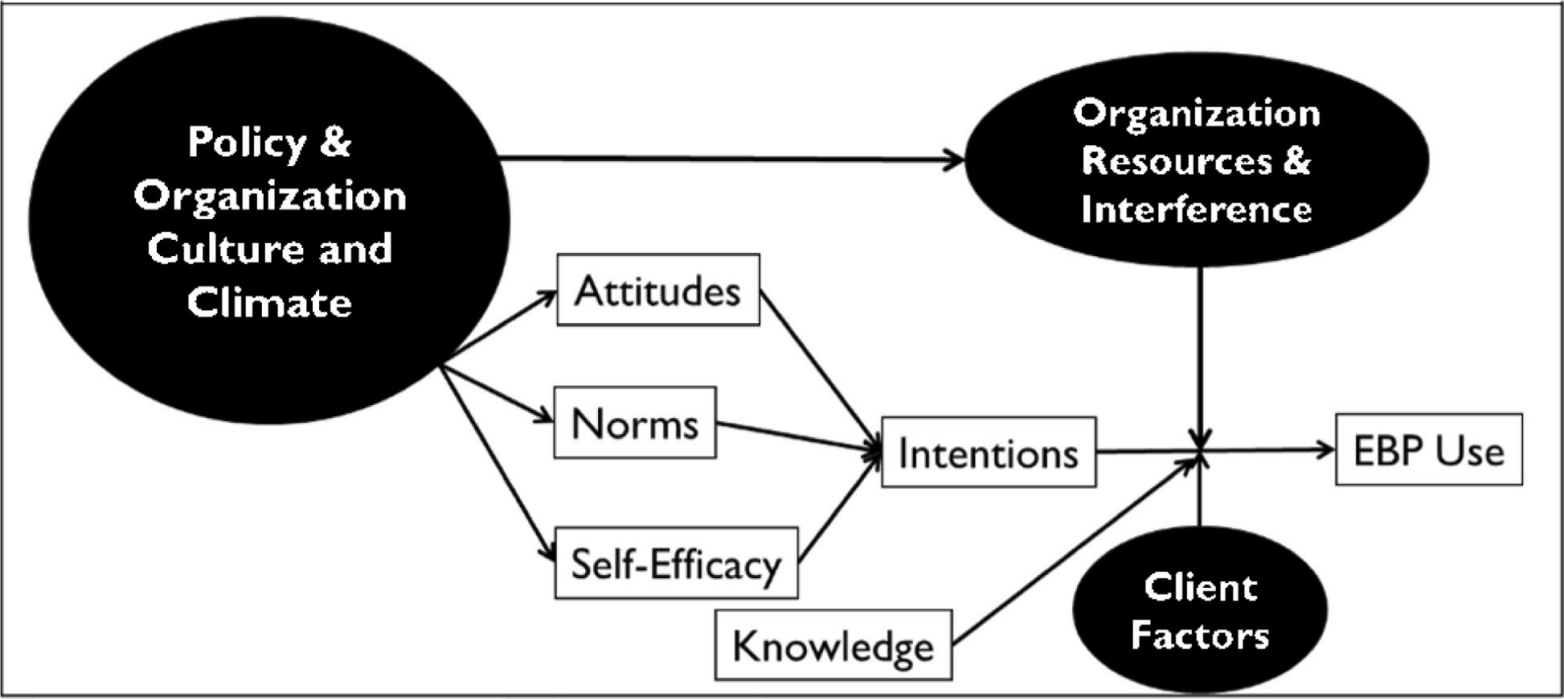
Causal model predicting EBP use. Note. Intentions and EBP use constitute our primary outcomes of interest. Attitudes, norms, self-efficacy, knowledge, and organizational culture and climate are conceptualized as antecedent factors to intention formation. Organizational resources, interference, and client factors are hypothesized to serve as potential moderators of intention to behavior

This study (Assessing Causal Pathways and Targets of Implementation Variability for EBP use; Project ACTIVE) will extend prior work by validating a causal model (see Fig. 1) to predict intentions to use and actual use of CBT components in a large sample of mental health clinicians. Studying intentions and clinician behavior (i.e., EBP use) is equally important with respect to understanding causal pathways of implementation. Research demonstrates that clinicians may not use a given EBP for one of two reasons: (1) either a clinician does not intend to use the component (i.e., has weak intentions) or (2) a clinician has strong intentions, but something interferes with their ability to act on those intentions. When intentions are weak, implementation strategies should target the underlying determinants of intention, with the goal of strengthening intention. When intentions are strong, but use is low, strategies should specifically be designed to help those who have already formed a strong intention to act on it.

As illustrated in Fig. 1, we predict that we will observe strong intentions to deliver CBT components when a clinician (a) perceives that their use of the component will be advantageous (attitudes), (b) perceives that administrators and supervisors expect them to deliver a CBT component and that other clinicians are delivering the component (subjective norms), and (c) believes that they can deliver the CBT component (self-efficacy). Knowledge can also play an important role in predicting behavior [21]; clinicians need to have the requisite knowledge of how to deliver each CBT component to act on their intentions.

Constructs from implementation science frameworks, which typically posit multilevel constructs that influence the implementation process [14, 22], are integrated in the model in two ways. First, we hypothesize that organizational factors (i.e., organizational culture, climate) function as *antecedents* to behavioral intention. Theory suggests that specific types of organizational culture and climate will act on clinicians who work in an organization to influence attitudes, establish norms, and influence self-efficacy with regard to the use of EBP, thereby increasing intentions to use EBPs [10, 23, 24]. For example, the effects of culture and climate may occur through an attraction-selection-attrition process, which refers to a bidirectional influence of organizational and clinician characteristics in which clinicians are attracted to certain organizations, organizations select to hire clinicians with an epistemological approach or background consistent with that organizations’ philosophy, which in turn solidifies organizational culture [25]. More specifically, an organization with a high level of EBP implementation climate (defined as an organization in which clinicians perceive that EBP use is highly prioritized, valued, expected, supported, and rewarded [26]) would be expected to exert normative pressure on a clinician to use EBP, foster positive attitudes toward EBP, and provide infrastructure and clinical support to bolster clinician self-efficacy in the use of EBP, thus leading to higher intentions to use a given EBP. Second, we hypothesize that additional contextual factors (e.g., organizational resources, clinician workload, client factors) may moderate the association between intention and behavior. For example, a prompt in an organization’s electronic health record may strengthen a given clinician’s ability to act on their intentions to review homework in a CBT session by reminding them to do so. Alternatively, a client presenting in crisis may interfere with a clinician’s follow-through on their intentions to use a CBT intervention by requiring them to attend to safety concerns.

CBT is an ideal intervention to use for a test of this model. CBT is a leading psychosocial intervention for many prevalent psychiatric disorders due to strong empirical support for its efficacy [27, 28] and cost-effectiveness [29, 30]. CBT has been a major target of system-wide implementation efforts over the past few decades [31–33], yet is rarely implemented with fidelity in community settings [2, 34]. CBT, like many leading psychosocial EBPs, also comprises many discrete components [35, 36] including both structural elements (e.g., agenda-setting, homework assignment) and intervention components (“strategies for change”) that comprise the protocol (e.g., cognitive restructuring, exposure). The use of components may vary based on the needs of the client. Thus, implementing a “single CBT protocol” requires clinicians to learn multiple components concurrently, which they use with varying fidelity [34, 37, 38]. Prior work has demonstrated that the strength of intentions varies by specific EBP component [20, 39]. There may be characteristics of specific EBP components (e.g., their salience, complexity) that influence the causal pathways that lead to implementation. Studying antecedents and moderators of intentions for diverse CBT components may point to levers that become part of tailored implementation strategies that vary for different EBPs.

Specific aims of this study are to:

- Aim 1: Test the extent to which intentions and their determinants (attitudes, self-efficacy, and subjective norms) differentially predict the use of six discrete components of CBT among a diverse group of clinicians.
- Aim 2: Test the pathways by which organizational factors predict determinants of intention and by which other contextual factors (e.g., client behaviors, clinician distress, caseload) can moderate the association between intentions and use of each CBT component.

## Methods/design

### Study setting

We will leverage ongoing CBT implementation efforts that the *Penn Collaborative for CBT and Implementation Science* (Penn Collaborative) leads in two large public health systems (Philadelphia and Texas) to recruit 300 mental health clinicians and 600 clients across 40 organizations. The Penn Collaborative is a clinical and research partnership with community behavioral health providers, payers, and networks. The Penn Collaborative has implemented transdiagnostic CBT in diverse public mental health settings across presenting psychiatric problems and levels of care [33, 40]. As of January 2020, the Penn Collaborative has trained 2048 clinicians in 159 community mental health programs. All clinicians trained through the Penn Collaborative are encouraged to use CBT interventions with appropriate clients on their caseload and are routinely engaged in data collection about their demographics and training backgrounds, knowledge of CBT, CBT skills, and other metrics as part of ongoing program evaluation. As such, the Penn Collaborative creates a natural laboratory within which to develop and test generalizable implementation strategies.

Clinicians trained through the Penn Collaborative are an ideal population for studying variability in intentions and use. First, all clinicians receive comparable initial training, yet prior work suggests variability in both intentions and use [39, 40]. Second, we can recruit study participants from two distinct implementation efforts in the city of Philadelphia and the state of Texas. We selected these locations because they vary in the extent to which organization leadership and payers are involved in the implementation processes. Thus, we expect substantial variation in our organization and clinician-level variables to support the test of the proposed causal model.

### Participants and procedures

We will use the same sample for aims 1 and 2. All participants will be recruited within organizations that are actively partnering with the Penn Collaborative following an individual therapist’s completion of initial CBT training activities through the Penn Collaborative. An overview of participant procedures and collected variables is detailed in Table 1. Clinicians will be eligible for the study if they have completed training through the Penn Collaborative and are proficient in English. Any client over the age of 7 who has a CBT session with these clinicians after they complete training is eligible to participate.

**Table 1:** Study timeline for participant procedures

	Study period				
Time point	Enrollment	Time 1 (post-training)	Time 2 (3 months post-training)	Time 3 (6 months post-training)	Time 4 (optional)
**Enrollment**					
Consent to contact	x				
Informed consent	x				
**Assessments**					
CBT Knowledge Quiz		x			
Clinician demographics		x			
CBT intentions		x	x	x	
CBT attitudes		x		x	
CBT norms		x			
CBT self-efficacy		x			
Implementation Climate Scale		x			
Organizational Social Context Measure		x			
Recorded therapy session coded with CTRS			x	x	
Organizational Resources			x	x	
Clinician Caseload			x	x	
Adapted TIB Scale			x	x	
CBT Use Self-Report			x	x	
Qualitative Interview					x

Note. *CBT* = Cognitive Behavioral Therapy, *CTRS* = Cognitive Therapy Rating Scale, *TIB* = Treatment Interfering Behavior

Recruitment procedures will vary between Philadelphia and Texas due to differences in program evaluation practices. In Philadelphia, prior to and immediately after initial training, clinicians complete electronic survey measures associated with the Penn Collaborative program evaluation. Both before and after completing these initial measures, they will receive brief informational materials about the study and be asked whether they would like to learn more from research staff. Research staff then will contact interested clinicians and describe participation in study activities. In Texas, we will obtain approval from clinics that are participating in the Penn Collaborative, and research staff will contact clinicians within 8 weeks post-training to participate. All participants will receive descriptions of the study procedures, which include (a) completing additional survey measures, which will take approximately 1 h (time 1); (b) granting permission for research staff to access two audio-recorded CBT sessions (which in Philadelphia are submitted as a part of program evaluation and in Texas will be collected by research staff); and (c) completing two brief, 10-min surveys electronically about their submitted audio-recorded therapy sessions when each recording is submitted. The first audio recording and brief survey will occur at approximately 3 months post-training (time 2) and the second at 6 months post-training (time 3). Trained study staff will obtain client consent (or caregiver consent and youth assent for clients under age 18) prior to session recordings.

All clinicians will be paid $50 for completing time 1 measures and $15 for each of the briefer time 2 and time 3 surveys. Philadelphia clinicians will not be paid for submitting their audio-recorded therapy sessions, as this activity is already required for Philadelphia clinicians participating in Penn Collaborative implementation efforts. Texas clinicians do not routinely submit audio recordings to the Penn Collaborative and therefore will be compensated $25 for recording each session; their clients will also be compensated $15 for allowing us to record their session. Research staff will supply audio recorders to participating Texas organizations for clinicians to record and upload their recordings through REDCap, a HIPAA-compliant data storage platform, using processes that have been used successfully in prior work [41]. To further incentivize recording, Texas clinicians also will receive a brief, 1–2 page feedback report from study staff on their CBT delivery, a clinician recruitment incentive which has been successful in several of our prior studies [42, 43]. Philadelphia clinicians already receive feedback from Penn Collaborative staff on their CBT delivery through their program evaluation participation. Procedures developed for Texas closely mirror those used in Philadelphia where rates of tape collection are quite high (~14 audio recordings per clinician).

At the conclusion of each project year, we will inspect the data to identify clinicians who report strong intentions (i.e., scores of 5 or higher out of 7 on an intentions scale; see *Measures* below) and low use on at least one of the six CBT components to purposively sample approximately 30 clinicians. We will invite these clinicians to participate in a 30-min semi-structured interview, during which we will present data about their intentions and use and inquire about which variables interfered with or inhibited their ability to act on their intentions to use CBT components. Clinicians will be compensated $30 for completing this interview. Based on our prior research, we anticipate easily identifying at least 30 clinicians who report strong intentions and demonstrate low use. For example, in a recently completed implementation trial, clinicians were asked to record sessions in which they plan to use CBT; however, direct observation suggests that approximately 20% of recorded sessions contained zero CBT components [42, 44].

### Measures: implementation outcomes of interest

We are interested in predicting clinician intentions and use of six specific CBT components: agenda setting, homework review, homework planning, cognitive restructuring, behavioral activation, and exposure therapy. We selected these to capture *structural* components of CBT (agenda setting, planning, and reviewing homework) that would apply to a broad client population regardless of the specific diagnosis, discrete *intervention* components that could apply either to a wide range of client presenting problems (cognitive restructuring), or used selectively for clients with common presenting problems (exposure therapy for anxiety or post-traumatic stress and behavioral activation for depression). While not an exhaustive list of CBT components, they are highly generalizable across a range of the most common mental disorders and vary considerably in their complexity, salience for specific presenting problems, and the preparation and resources required to implement them, making them ideal for study objectives.

#### CBT intentions

We will measure the strength of clinicians’ intentions to use each CBT component using established item stems from social psychology. These stems were designed to be adapted to any behavior of interest [18] and have been used successfully in implementation studies [20, 45]. Specifically, intention is measured using two items on a 7-point scale asking how willing and how likely one is to use each of the six CBT components of interest. Both response options use a 7-point scale, with higher scores representing stronger intention. For each component, the two measures will be averaged.

#### CBT use

The use of each CBT component will be measured through observation of their audio-recorded session using select items on the Cognitive Therapy Rating Scale (CTRS), the gold standard outcome metric used in the Penn Collaborative [40]. The CTRS is an 11-item observer-rated measure designed to evaluate clinicians’ CBT delivery [46]. Each item is scored on a 7-point Likert scale, ranging from 0 (*poor*) to 6 (*excellent*). The 11 items are summed to yield a total CTRS score from 0 to 66. Items measure General Therapy Skills (feedback, understanding, interpersonal effectiveness, collaboration), CBT Skills (guided discovery, focus on key cognitions and behavior, strategy for change, application of CBT techniques), and Structure (agenda, pacing and efficient use of time, use of homework assignments) [47, 48]. The CTRS has demonstrated internal consistency and inter-rater reliability [46] and strong inter-rater agreement for general competency [49].

Three CTRS items will be our primary outcomes of interest: “Agenda,” “Homework,” and “Strategy for Change,” with “use” defined as an item score of 3 or higher. “Agenda” indicates the extent to which the therapist collaboratively set a specific plan with the client at the beginning of the session. “Homework” indicates the extent to which the therapist worked with the client to review the client’s planned practice of skills since the last session, and collaboratively identify additional practice to be completed before the next session. “Strategy for Change” indicates the extent to which the therapist selected appropriate CBT strategies for use in the session. Of note, the standard CTRS does not delineate specific “strategies for change” to be coded. We will modify the CTRS slightly for the purposes of this study: coders will denote a specific “strategy for change” in addition to providing the overall score. Coders will receive definitions for each of the “strategies for change” of interest (exposure, behavioral activation, and cognitive restructuring). We will follow standardized, rigorous procedures for ensuring inter-rater reliability and conduct weekly coding meetings to prevent coder drift. Additionally, 20% of all sessions will be double-coded.

### Measures: causal model inputs of antecedents to intention (administered at time 1)

#### Attitudes

Attitudes toward each of the six CBT components will be measured using two standard semantic differential scales [11, 50]. Respondents will use 7-point scales ranging from “extremely unpleasant” to “extremely pleasant” and from “extremely useless” to “extremely useful” (with these anchors scored −3 to +3). A total score will be computed by averaging all *z*-standardized items, where the higher the score is, the more favorable the attitude.

#### Norms

We will measure norms for each of the six CBT components through two standard item stems regarding the perception that other people like them (i.e., other clinicians) will use the component and that other people who are important to them (e.g., clinical supervisor) will approve of them using the component. For each statement, respondents will rate the extent to which they agree on a 7-point scale from “strongly disagree” to “strongly agree.”

#### Self-efficacy

We will measure self-efficacy for each of the six CBT components through responses to four statements on a 7-point scale from “strongly disagree” to “strongly agree.” This scale will measure clinicians’ perceptions of their skills and abilities to deliver each CBT component. For example, respondents will be asked to rate their agreement with the statement “If I wanted to, I could use cognitive restructuring with each of my clients receiving CBT.”

#### Knowledge

The Cognitive Therapy Knowledge Quiz [40] is a brief knowledge questionnaire that is administered as part of all Penn Collaborative Training efforts. Administered at post-training (which aligns with study time 1), this knowledge measure comprises 20 items that assess a clinicians’ general knowledge of CBT. This measure has adequate psychometrics and demonstrated sensitivity to change following CBT training [40].

#### Organizational culture and molar organizational climate

Organizational culture and climate are abstractions that describe the shared meanings that organizational members attach to their work environments [51, 52]. Here, organizational culture refers to clinicians’ shared perceptions of the collective norms and values that characterize and guide behavior within an organization [53]. Molar organizational climate refers to clinicians’ shared perceptions of the psychological impact of the work environment on their individual well-being [54]. The Organizational Social Context Measurement System (OSC) is a well-validated measure developed specifically to assess organizational culture and climate in mental health and social service organizations [53, 55]. The OSC has an established factor structure that indexes both organizational culture and molar climate. It has established national norms and has strong evidence of reliability and validity [53, 56]. This measure yields six subscales of interest for this study: three indexing facets of organizational culture (proficiency, rigidity, and resistance) and three indexing molar organizational climate (engagement, functionality, and stress).

#### Implementation climate

Implementation climate is defined as clinicians’ shared perceptions of the extent to which the use of an EBP is expected, supported, and rewarded within an organization [23, 57]. In this study, we will assess the implementation climate for CBT use specifically. The 27-item Implementation Climate Scale (ICS [22];) total score will measure implementation climate for CBT use. The ICS assesses several facets of implementation climate, including organizational focus on CBT, educational support for CBT, recognition for using CBT, rewards for using CBT, selection (i.e., hiring) of staff for CBT, and selection of staff for openness. The ICS has demonstrated good reliability and validity [22], a stable factor structure [58], and associations with EBP use in prior research [59].

Both the OSC and ICS will be collected by a representative number of clinicians (minimum of 3) within each organization. Measures are completed by individual clinicians and will be aggregated to the organizational level, following evaluation of inter-rater agreement and other indices of construct validity, in accordance with recommended practices [60].

### Measures: causal model inputs of moderators of intention to behavior (administered at time 2 and time 3)

#### Organizational resources

We adapted two measures that include assessments of organizational resources [61, 62] to create a brief assessment of organizational resources which may facilitate the delivery of each of the six CBT components. For example, items will ask about access to a printer to facilitate the use of worksheets in session and whether the clinician has dedicated time outside of scheduled sessions to prepare for CBT delivery.

#### Clinician workload

We will ask clinicians to report on their current clinical caseload as a proxy for their current workload.

#### Client factors

We will assess client demographics (age, gender, race, ethnicity), the primary problem(s) for which the client is seeking treatment, and the number of CBT sessions attended previously from the treating clinician. We also will assess client behaviors that may interfere with a clinician’s ability to act on intentions to deliver CBT in session by adapting the Treatment-Interfering Behaviors (TIBs) Checklist [63]. TIBs are defined as any behaviors that negatively interfere with a client’s ability to successfully engage in treatment. The TIBs Checklist was established to measure behaviors clients engage in that interfere with the therapy process proceeding as intended (e.g., showing up late, presenting in crisis, making threats, refusal to engage) across a course of treatment. We adapted this measure to assess for session-level barriers that may inhibit CBT delivery. Clinicians will be asked to report which (if any) behaviors the client exhibited in that session.

#### Qualitative interviews

We have less scientific understanding of variables that may moderate the intention to behavior gap for CBT implementation than we do of antecedents to intention formation. To gain a richer understanding of this causal pathway, we will conduct brief interviews among 30 purposively sampled clinicians who report strong intentions but low use of at least one CBT component. During this interview, clinicians will be presented with a transcript of their recorded therapy session to remind them of their performance, along with their reported initial intentions to use each of the CBT components. A semi-structured interview will ask clinicians to reflect generally on their perceptions of what may have interfered with their intended use of CBT intervention components. A series of follow-up probes will ask clinicians about their perception of whether variables identified in the model (organizational resources, workload, client factors) may affect their ability to implement each of the six CBT components of interest. Probes will ask about characteristics of each of the six intervention components, and more general organizational factors (e.g., “what other factors in your organization do you believe made it challenging to act on your intentions to deliver X intervention”). Interviews will be audio recorded and transcribed.

#### Covariates

To control for differences across sites in workforce composition and potential confounds in our causal models, we will collect information from the Penn Collaborative program evaluation measures about clinician demographics (age, race, ethnicity, gender) and clinical background (educational background, years of clinical experience, licensure status, primary clinical responsibilities, theoretical orientation, prior experience with CBT, and amount of supervision received).

### Data analysis plan

Due to the clustering of clinicians within organizations, we will use multilevel path analysis (ML-PA) as our general analytic framework [64, 65]. ML-PA is a special case of multilevel structural equation modeling that incorporates random intercepts and allows investigators to specify separate structural models to account for variance in level 1 dependent variables at each of the nested levels. We will use a two-level ML-PA with clinicians (level 1) nested within organizations (level 2). Analyses will be implemented via the Muthén and Asparouhov algorithm in Mplus [66] which accommodates missing data and unbalanced cluster sizes, incorporates a robust maximum likelihood estimator (MLR) that does not require a strict normality assumption, and generates robust estimates of standard errors and chi-square [67].

#### Preliminary analysis

We will examine the psychometric properties of total scales and subscales of all measures (e.g., coefficient alpha, confirmatory factor analyses). We will examine bivariate associations and transform the data as appropriate. Data missing at random will be modeled using full information maximum likelihood estimation. We will confirm the construct validity of all compositional organization-level variables (i.e., culture/climate) by examining within-organization agreement (e.g., rwg, awg) and between-organization variance (e.g., eta-squared, ICC(1)) prior to aggregation [68–72]. For the OSC, calculation of organization-level scores will be done by the OSC development team; OSC scores will be normed based on a national sample of mental health clinics [53].

#### Aim 1 analysis

This analysis will identify the relative contribution of clinicians’ attitudes, self-efficacy, and normative pressure to each CBT component, to explain variation in intention and use of each component at level 1. The analysis will determine if a homogenous or heterogeneous set of factors influence intentions to use each EBP component. We will conduct separate ML-PA models for each of the six CBT components. Because self-efficacy, attitudes, and subjective norm affect clinician behavior indirectly through intention, models will be overidentified, thus permitting tests of model fit. We will test model fit for each CBT component using model test statistics (e.g., model chi-square), information criteria (e.g., BIC), and more focused indices of approximate fit (e.g., RMSEA, CFI, SRMR), including examination of standardized beta coefficients and residuals to identify specific points of suboptimality [73].

We will leverage the fact that we have two observations of CBT use per clinician to (1) test our model on clinicians’ initial CTRS score for each of the six CBT components of interest to determine model fit and parameter values and then (2) validate and confirm our final model on a hold-out sample consisting of clinicians’ second CTRS score for the same CBT component. In the confirmatory hold-out analysis, we will fix model path parameters to values obtained in the initial analysis and then test model fit; this will allow us to test whether the parameter values are generalizable. Should good model fit be attained in the second confirmatory analysis, we will have high confidence in the generalizability of our causal model. Should this confirmatory analysis yield poor model fit, we will systematically release (i.e., freely estimate) path coefficients to determine which pathways are generalizable and which are not. To test whether determinants of intention and behavior are comparable across the six CBT components, we will additionally conduct *z*-tests that compare the magnitude of the path coefficients linking attitudes, self-efficacy, and subjective norms to intentions across the component models and we will report the *p*-values associated with these tests. The *z*-tests will be calculated as *z* = *B*_coefficient1_ − *B*_coefficient2_/√(*SE*^*2*^_*coefficient1*_ + *SE*^*2*^_*coefficient2*_) [74]. This will formally test whether specific antecedents (attitudes, self-efficacy, norms) are differentially related to intention across components.

#### Analysis of potential covariates

Demographic and other clinician characteristics, such as years of experience, will be tested as covariates by including them in linear equations associated with all endogenous variables in the model, the use of the component, and the intention to use that component. This approach controls for potential confounds and spurious effects, while minimizing the impact of the outcome variance explained by covariates rather than the predictors of interest. Covariates used in aim 1 will be carried forward into aim 2 analyses.

#### Aim 2 analysis

Aim 2 analyses comprise both a quantitative and a qualitative and mixed-methods approach. We first will test the extent to which hypothesized antecedent organizational factors at level 2 are associated with organization-level variance in attitudes, self-efficacy, and norms; intention; and use of the CBT components. Building on our aim 1 model described above, we will add organizational variables (organizational culture, molar climate, and implementation climate) at level 2 to estimate pathways between organizational variables and the organization intercepts of attitudes, norms, and self-efficacy, as pictured in Fig. 1. Given competing theoretical models of organizational culture and climate and the mixed state of the empirical literature linking these constructs to CBT use, we will systematically test a series of hypothesized models to determine which level 2 model best accounts for organization-level variance in attitudes, norms, and self-efficacy to use each CBT component. The first set of models will use a single dimension of culture, molar climate, or implementation climate to predict level 2 variance in attitudes, self-efficacy, and norms. The second set of models will compare a simultaneous model to a sequential model for organizational antecedents. The simultaneous model is one in which culture, molar climate, and implementation climate are all entered simultaneously as antecedents to attitudes, self-efficacy, and norms. The sequential model will position culture as an antecedent to molar climate and implementation climate, with climate scores subsequently influencing attitudes, self-efficacy, and norms. Similar to aim 1, we will determine the optimally fitting model based on model test statistics (e.g., model chi-square) as well as indices of approximate fit (e.g., RMSEA, CFI, SRMR) and examination of standardized beta coefficients and residuals [73, 75]. We will also examine model *R*^2^ to determine which model is optimally predictive of targeted outcomes.

We again will leverage the fact that we have two observations per therapist to validate our model at level 2 by (a) identifying the best fitting level 2 model using therapists’ first observation and then (b) re-estimating the entire model on their second observation with fixed paths to assess the generalizability of the path coefficients. If we attain good model fit in the second confirmatory analysis, this suggests that our level 2 causal model is highly generalizable. If the confirmatory analysis yields poor model fit, we will systematically release (i.e., freely estimate) path coefficients to determine which pathways are generalizable and which are not. Results will provide some of the first tests linking organizational culture, molar climate, and implementation climate to observed CBT use and will offer evidence regarding the generalizability of these relationships across CBT components.

Once we have determined the optimal structure for the organizational antecedents, we will test the moderating effect of organizational resources, clinician workload, and client factors on the association between intention and use of each CBT component, by incorporating interaction terms between intention and each of the hypothesized moderating variables predicting CBT component use at level 1.

Qualitative analysis of interviews will supplement quantitative results to help us understand possible moderators of the intention to behavior gap. Analysis will be guided by an integrated approach [76] which uses an inductive process of iterative coding to identify recurrent themes, categories, and relationships. We will develop a structured codebook and code for a priori attributes of interest (i.e., the role of organizational resources and client factors as possible points of interference for acting on intentions) and also use modified grounded theory [77], which provides a systematic and rigorous approach to identifying codes and themes. Using a qualitative data analysis software program, two members of the research team will separately code a sample of 3 transcripts and compare their application of the coding scheme to assess the reliability and robustness of the coding scheme. Any disagreements will be resolved through discussion. The team will refine the codebook as needed. Coders will be expected to reach and maintain reliability at *κ* ≥ .85. After coding is complete, the team will read through all codes to examine themes and produce memos of examples and commentary. We will use mixed methods to analyze themes as a function of clinician and organizational characteristics to identify patterns of responding. First, we will use findings from quantitative data to identify patterns in the qualitative data by entering quantitative findings into our software as attributes of each participant. Quantitative attributes will be used to categorize and compare important themes among subgroups. For example, we may enter organizational implementation climate scores and categorize clinicians into three groups: those working in low climate, average climate, and high climate settings. Then, if organizational resources emerge as a theme from the interviews, we can query instances when organizational resources are discussed in low, average, and high climate environments. We will then identify patterns and make interpretations across groups based on quantitative categorization.

#### Power analysis

We used a conservative, formula-based approach to determine the necessary sample size to test our ML-PA model at levels 1 and 2 [78, 79]. First, we used power formulas for regression/path analysis in combination with a design effect (to account for the non-independence of clinicians nested within organizations) to estimate the level 1 sample size necessary to detect a small effect (incremental *R*^2^ = .03) of a single predictor in our hypothesized path model. Assuming alpha = .05, *K* = 40 organizations (based on feasibility and budget), *n* = 3 to *n* = 7 clinicians per organization (based on pilot data), and an intraclass correlation coefficient (ICC) of our dependent variables (e.g., intention, use) ranging from .05 to .20 (based on our preliminary studies [59]), we need a level 1 sample size of 300 clinicians to achieve a power of .8 to .9. Based on these calculations, we set our level 1 sample size at *N* = 300 clinicians. We then calculated the minimum detectable effect size (MDES) for a single predictor at level 2 of our ML-PA model assuming a fixed sample size of *K* = 40 organizations, alpha = .05, and power ranging from .7 to .9. Given these parameters, we will be able to detect individual path effects of size *f*^2^ = .16 to .28, which fall within the medium-to-large range [78]. Our preliminary studies indicate this is a reasonable range of MDESs at level 2 given that organizational variables as a group routinely account for ≥ 70% of the level 2 variance in implementation antecedents and outcomes. Based on these analyses, the proposed sample size of *N* = 300 clinicians nested within *K* = 40 organizations is sufficiently large to provide a robust and sensitive test of the hypothesized ML-PA models.

## Discussion

Causal modeling of implementation processes holds great promise for advancing our ability to generate effective and efficient implementation strategies. This study will empirically test a promising causal model in a large sample of mental health clinicians trained in CBT, a leading mental health EBP. It will be one of the first studies to test a highly specified causal model predicting the use of discrete EBP components. Outcomes will have a significant impact on the advancement of causal theory in implementation science by identifying mechanistic pathways of implementation for diverse EBP components. Identifying the exact pathways by which implementation occurs will provide insights into how to generate tailored and effective implementation strategies across the multilevel constructs implicated in the implementation process. As detailed in Table 2, implementation strategies to address distinct constructs delineated in the causal model could look quite different. For example, if low CBT use is driven by poor attitudes about its use, direct financial incentives to the clinician may be warranted. If low use is driven by norms, then clear organizational directives or leaderboards comparing use among clinicians would be more effective.

**Table 2:** Examples of implementation strategies implicated by identified causal mechanisms

Model target	Examples of implicated implementation strategies
Knowledge	Education/training
Attitudes	Financial incentives
Norms	Policy mandates, leaderboards
Self-efficacy	Supervision enhancement, role-plays
Organizational culture	Work-environment interventions (e.g., Availability, Responsiveness, and Continuity; ARC) [80]
Organizational climate	Work-environment interventions (e.g., Availability, Responsiveness, and Continuity; ARC) [80]
Implementation climate	Targeted organizational development strategies (e.g., Leadership and Organizational Change for Implementation; LOCI) [81]
Resources	Financial investment, resource banks
Interference	Reminder prompts in electronic health records

Of note, our guiding causal model does not make specific hypotheses regarding where in the causal chain the organizational variables of interest lie. We considered making stronger hypotheses about which organizational factors may be more proximally related to attitudes, norms, and self-efficacy. However, given the development of multiple competing streams of empirical literature examining cross-level effects in implementation studies, we decided to retain an equivocal approach and test several alternative models to advance the literature in this area. We also considered measuring other organizational constructs highlighted in the implementation literature, such as implementation leadership, which is theorized to precede the development of implementation climate. Ultimately, we selected the constructs that represent the most parsimonious set of organizational variables and would minimize respondent burden.

Outcomes from this study also will yield critical insights as to whether a single implementation strategy can increase the use of a complex psychosocial intervention, or whether different strategies are needed for different components of the EBP. For example, in CBT, increasing agenda setting may require strategies that help clinicians remember to act on their strong intentions to set an agenda, whereas increasing the use of exposure may require supports that increase self-efficacy. Relatedly, findings from our study will set the stage for future research that tailors implementation strategies to specific intervention characteristics (e.g., complexity, salience). Findings will inform studies that compare the effectiveness of implementation strategies targeting identified mechanisms to improve the rates with which EBPs are delivered in community settings. Ultimately, this study will inform efforts to reduce psychiatric burden and alleviate suffering of those with mental illness.

## Conclusions

Successful completion of this study will advance implementation theory, currently in its infancy, and identify a set of malleable targets for implementation strategies. By leading to more efficient and effective implementation strategies, findings will inform future research aimed at improving rates of EBP use in community settings, alleviating the suffering of those with mental illness.

## Data Availability

Not applicable.

## Abbreviations

EBP: Evidence-based practice
CBT: Cognitive-behavioral therapy
OSC: Organizational Social Context
ICS: Implementation Climate Scale
TIB: Treatment Interfering Behavior
